# Structural and Evolutionary Adaptations of Nei-Like DNA Glycosylases Proteins Involved in Base Excision Repair of Oxidative DNA Damage in Vertebrates

**DOI:** 10.1155/2022/1144387

**Published:** 2022-04-04

**Authors:** Hafiz Ishfaq Ahmad, Gulnaz Afzal, Sehrish Sadia, Ghulam Haider, Shakeel Ahmed, Saba Saeed, Jinping Chen

**Affiliations:** ^1^Department of Animal Breeding and Genetics, University of Veterinary and Animal Sciences, Lahore, Pakistan; ^2^Department of Zoology, The Islamia University of Bahawalpur, Bahawalpur, Pakistan; ^3^Department of Biological Sciences, University of Veterinary and Animal Sciences, Ravi Campus, Pattoki, Pakistan; ^4^Instituto de Farmacia, Facultad de Ciencias, Universidad Austral de Chile, Campus Isla Teja, 5090000 Valdivia, Chile; ^5^Institute of Physics, The Islamia University of Bahawalpur, Bahawalpur, Pakistan; ^6^Guangdong Key Laboratory of Animal Conservation and Resource Utilization, Guangdong Public Laboratory of Wild Animal Conservation and Utilization, Institute of Zoology, Guangdong Academy of Sciences, Guangzhou, Guangdong, China

## Abstract

Oxidative stress is a type of stress that damages DNA and can occur from both endogenous and exogenous sources. Damage to DNA caused by oxidative stress can result in base modifications that promote replication errors and the formation of sites of base loss, which pose unique challenges to the preservation of genomic integrity. However, the adaptive evolution of the DNA repair mechanism is poorly understood in vertebrates. This research aimed to explore the evolutionary relationships, physicochemical characteristics, and comparative genomic analysis of the Nei-like glycosylase gene family involved in DNA base repair in the vertebrates. The genomic sequences of NEIL1, NEIL2, and NEIL3 genes were aligned to observe selection constraints in the genes, which were relatively low conserved across vertebrate species. The positive selection signals were identified in these genes across the vertebrate lineages. We identified that only about 2.7% of codons in these genes were subjected to positive selection. We also revealed that positive selection pressure was increased in the Fapy-DNA-glyco and H2TH domain, which are involved in the base excision repair of DNA that has been damaged by oxidative stress. Gene structure, motif, and conserved domain analysis indicated that the Nei-like glycosylase genes in mammals and avians are evolutionarily low conserved compared to other glycosylase genes in other “vertebrates” species. This study revealed that adaptive selection played a critical role in the evolution of Nei-like glycosylase in vertebrate species. Systematic comparative genome analyses will give key insights to elucidate the links between DNA repair and the development of lifespan in various organisms as more diverse vertebrate genome sequences become accessible.

## 1. Introduction

DNA oxidative damage occurs due to various factors, including external agents, endogenous oxygen species formed during normal cellular respiration, and other essential activities, such as demethylation, that generate intermediates, including basic sites [1]. Incorrectly handled or repaired sites might cause polymerase to halt replication, eventually leading to mutagenesis and cancer [2]. Mammalian cells utilize a battery of enzymes geared at repairing DNA damage to ensure the genome's accurate replication and transcript interpretation [3]. The base excision repair (BER) process is initiated by several glycosylases, which safeguard cells against mutagenesis and oxidative DNA damage by identifying damaged bases and commencing the repair process [4]. This pathway is the primary method for eliminating oxidative DNA damage from the genome, making it an important step in ensuring genome integrity in the first place. The prevention of disorders induced by oxidative DNA damage is therefore dependent on the function of this system [5]. To eliminate oxidative DNA base damage in mammalian cells, at least five distinct DNA glycosylases with overlapping substrate specificities are required, each with a different substrate specificity [6]. The Nei-like DNA glycosylases (NEIL1/2/3), 8-oxoguanine glycosylase 1 (OGG1), and endonuclease three homologue 1 (NTH1) are only a few of the enzymes that exist [7]. One of the functions of NEIL1 is to repair DNA that has been impaired by mutagenic chemicals or oxidation. A DNA glycosylase is an enzyme that detects and eliminates impaired bases from DNA strands. It is particularly favorable to oxidize pyrimidines such as formamidopyrimidine (Fapy), 5-hydroxyuracil, and thymine glycol [8]. Oxidative damage to DNA may be generated in various ways, including endogenously created oxygen species from normal cellular respiration and other critical operations such as demethylation, which form intermediates that include abasic sites [1, 9]. If the polymerase cannot complete its replication cycle due to insufficient site management or repair, this might result in mutagenesis and cancer in the long run [1]. In addition to excising an oxidized base (glycosylase activity), this bifunctional enzyme, according to the researchers, is capable of cutting the DNA backbone (lyase activity), and it can function independently of apurinic endonuclease (APE) [10]. Both double-stranded DNA (dsDNA) and single-stranded DNA (ssDNA) are targets for NEIL2, which has a bias for bubble DNA structures as a substrate and a preference for cytosine oxidation products as an enzyme [11]. Despite their considerable sequence variety, the Fpg/Nei family of proteins exhibits exceptional structural conservation of the two-domain design, notably in the N-terminal domain, largely composed of b-strands and are substantially maintained. C-terminal domain of the protein (which is rather stable across the whole protein family) comprises DNA-binding motifs such as the two-turn helix (H2TH) and the zinc or zincless finger (ZFF) [12].

The study of repair in diverse species can also allow researchers better understand how repair proteins and processes have evolved through time. This is important because the repair is an important biological function and because understanding how repair develops may help direct comparative repair research [13]. Generally, the evolutionary approach to comparative study is useful because it allows researchers to focus on how and why similarities and differences have arisen rather than merely detecting and categorizing them [14]. We feel that understanding the changes in DNA repair between species and the mechanisms and roles of particular DNA repair processes requires an evolutionary viewpoint in DNA repair research [15].

Unfortunately, comparative and evolutionary investigations of DNA repair mechanisms have been limited by a lack of thorough studies across a wide variety of species representing a wide range of ecological and evolutionary heterogeneity [13]. Recently, whole-genome sequences have been identified to represent a potentially helpful new source of comparative repair data. Entire genome sequencing is predicted to enable the prediction of a strain's or species' phenotype and the collection and analysis of a large amount of data for comparative study [16]. However, extracting useful information from complete genome sequences is quite challenging in practice. Despite the enormous number of studies completed in animals, no comparative genome analysis studies in vertebrates have been conducted to find the DNA repair genes linked to the development of long life [17]. According to current studies, all critical DNA repair pathways are low conserved in mammals [18].

Additionally, many nonmodel vertebrate genomes are becoming available for analysis. There is evidence that stress contributes to major selective factors driving the evolution. Numerous new genomes now enabled evaluations across different lineages to ascertain how certain genes are exposed to positive selection [19]. Pathogens are also the principal selective factor driving evolution. Numerous additional genomes now enable comparisons across lineages to ascertain the extent to which certain genes are exposed to negative selection [20].

The purpose of this study is to examine the evolutionary connection, physiochemical features, and comparative genomics of Nei-like DNA glycosylases gene family in the vertebrate species. We used more than 153 species of vertebrates, including mammalian, avian, and amphibian species, to conduct the systematic comparative analyses of the Nei-like DNA glycosylase genes encoding protein that regulate the DNA repair process in the vertebrate species with various life forms, including long-lived mammalian, avian, and amphibian species. This study examined the genomic sequences of Nei-like DNA glycosylases in the vertebrate species to determine the selection pressure exerted on these genes, which might be important in the adaptive evolution process. In this study, we explore how these genes evolved in various vertebrates and how selection and diversity influenced the evolution of this gene family over the years.

## 2. Materials and Methods

### 2.1. Data Collection and Sequence Analysis

Ensembl and NCBI databases were used to retrieve the genomic sequences of Nei-like DNA glycosylase genes (NEIL1, NEIL2, and NIL3) in the vertebrate species. To extract nonredundant protein sequences, the human Nei-like DNA glycosylase protein sequences (ENSG00000140398, ENSG00000154328, and ENSG00000109674) were utilized in a BLAST search [21]. The accession numbers of these genes were used to search the NCBI and Ensembl databases for coding sequences (CDS) of vertebrate species (Supplementary Tables S1-S3). Furthermore, using tBLASTn and BLASTn searches, orthologs in the vertebrate genome were recognized [22]. The OMA v.1.0.0 tool also analyzed homology patterns across protein-coding genes throughout the sequenced vertebrate species [23]. MAFFT v.7.221 [24] was used to align the sequences. Further analyses were conducted using these aligned sequences of homologous proteins.

### 2.2. Sequence Alignment and Test for Selection

The PROBCONS version 1.12 was used to create sequence alignments [25]. Gblocks v0.91b [26] was used to identify and screen possibly inaccurate and misaligned sections in the alignments utilized for phylogenetic analysis. MrBayesv3.2.2 (http://mrbayes.csit.fsu.edu/) [27] was used to create a phylogenetic tree to find the positive selection in each codon of NEIL1, NEIL2, and NEIL3 genomes. In Mrbayes, all analyses were done with a generation of 2000,000. With split frequency values of less than 0.01, convergence was believed to have been identified. This continued with further 2000,000 generations if the split frequency did not decrease below 0.01.

Positive selection signatures were identified by comparing the ratio of sites in positively selected codons across vertebrate species to the nearly neutral evolution model. Sites having greater nonsynonymous-synonymous substitution ratios were recognized as positively selected sites [28]. The ratios of dN/dS per site were examined using maximum likelihood approaches to evaluate positive selection at NEIL genes. Positive selection is shown by a dN/dS ratio greater than one. We employ two machine learning frameworks to increase the rigor of the positive selection: the PAML codeml program [29] and the Datamonkey Web Server's HyPhy package (http://www.datamonkey.org) [30]. Two different models (M7 vs. M8) were preferred and compared using log-likelihood values (2lnL) to determine if positive selection was acting on locations within each NEIL gene. The Bayes empirical Bayes (BEB) approach was combined with site models to determine which codons in codeml are flexible [29]. We recognized sites with a *p* value of 0.05 for SLAC and FEL and a Bayes factor 90 for REL to identify candidates for selection. The term “adaptive protein-coding substitutions” refers to those found using two or more machine learning algorithms [31]. Only locations that demonstrated selection signals in at least two of the machine learning approaches were examined to identify robust areas subject to positive selection. We examined codon positions under selection pressure in aligned sequences of targeted genes using Selecton version 2.2 (http://selecton.tau.ac.il/) [32]. As determined by the maximum likelihood value calculated by Bayesian inference [33], Selecton allows the ratio to vary between unique codons within a multiple sequence alignment. Furthermore, the outcomes of Selecton tool were graphically represented using color scales that depicted the various types of selections performed [34].

### 2.3. Structural Analysis and Homology Modeling

We evaluated and compared models to predict protein secondary and tertiary structure using the Phyre2 server [35] and Chimera 1.11.2 [36]. To find protein functional domains, we used the HMMER v3.1 [37], NCBI Conserved Domain Database [38], and Pfam v35.0 tool [39]. We used DOG 1.0 illustrator of protein domain structures [40] to illustrate protein domains. A simple and graphic illustration of protein domain structures with functional motifs assists a vast readership in quickly comprehending the ancient and novel functions of proteins.

### 2.4. Conservation Analysis

The ConSurf server (http://consurf.tau.ac.il/) [41] was used to examine the evolutionary conservation of amino acid residues in the human NEIL1, NEIL2, and NEIL3 proteins. Protein amino acids are more conserved than other amino acids because they are required for protein networks or are positioned in more enzymatic locations. As a result, alterations in conserved amino acids are more detrimental to protein function and structure than polymorphisms in flexible protein areas [41]. The conservation values ranging from 1 to 9 were used to predict conserved amino acids; conservation values between 1 and 4 are considered variable; 5–6 indicate moderate conservation; and 7–9 indicate very high conservation [41, 42].

### 2.5. Network and Coevolution Analysis

The CoeViz 2, a web-based program [43], was used to conduct a targeted and quick assessment of protein features, such as structural and functional regions, and give a variable analysis and visual representations of paired coevolution of amino acids. We used CoeViz analysis with two covariance metrics [44] to obtain the complete protein sequences of the proteins NEIL1, NEIL2, and NEIL3, which were used for phylogenetic analysis to assess the positions of covarying sites and large coincides with domains of the protein and used circular visuals for the understanding of residue interactions. The coevolving sites were emphasized in three-dimensional structures of protein sequences. Metascape (https://metascape.org/) [45] was also used to analyze the data for gene ontology (GO) and the Kyoto Encyclopedia of Genes and Genomes (KEGG) [46]. Drug-target-pathway networks were created using Cytoscape [47].

## 3. Results

The purpose of this study was to evaluate the gene sequences of Nei-like glycosylase in different “vertebrates” species to estimate the selection vigor in these genes, which may be involved in adaptive evolution. We explored the vertebrate genome for three genes involved in repairing DNA bases initiated by a series of DNA glycosylases such as NEIL1, NEIL2, and NEIL3, damaged by reactive oxygen species. We identified that these genes show the signature of selection during adaptive evolution which involved repairing of DNA bases damaged by reactive oxygen species. Maximum likelihood and phylogenetic analysis of coding sequences from 157 distinct vertebrate species revealed that the NEIL gene family descended from a common vertebrate ancestor. The evolutionary processes, phylogenetic linkages, structural and functional constraints of the NEIL1 and NEIL2 homologs in the vertebrate species, and their structural and functional constraints, were investigated in this study.

### 3.1. Protein Domains and Selection Analysis

**Figure 1:**
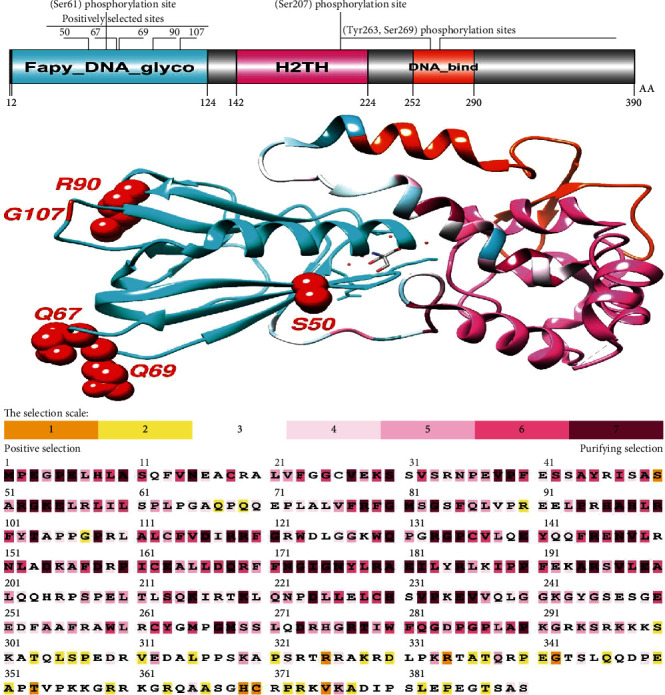
Illustration of protein domain structures using DOG 1.0 illustrator. The phosphorylation sites of the NEIL1 protein are shown in the molecular structure and conserved domain analyses. Positively selected amino acid sites were found in conserved domains, especially the H2TH domain. Positively identified locations were drawn into the 3D structure using human NEIL1 as a reference. Selecton analyses of human NEIL1 are color-coded and compared to sequences from aligned nucleotide coding sequences. Yellow and brown highlights represent positive selection, the neutral selection is represented by grey and white highlights, and purple highlights on codons represent purifying selection.

**Figure 2:**
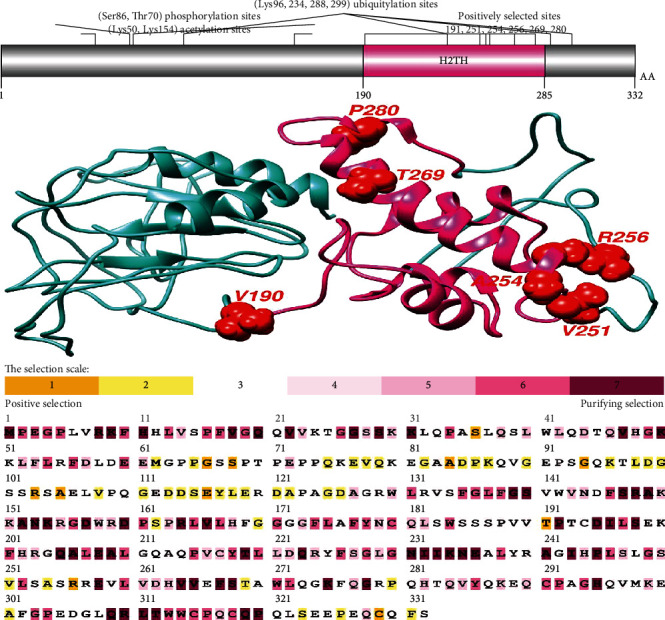
Illustration of protein domain structures using DOG 1.0 illustrator. The phosphorylation and ubiquitylation sites of the NEIL2 protein are shown in the molecular structure and conserved domain analyses. Positively selected amino acid sites were found in conserved domains, especially in the H2TH domain. Positively identified locations were drawn into the 3D structure using human NEIL2 as a reference. Selecton analyses of human NEIL2 are color-coded and compared to sequences from aligned nucleotide coding sequences. Yellow and brown highlights represent positive selection, the neutral selection is represented by grey and white highlights, and purple highlights on codons represent purifying selection.

**Figure 3:**
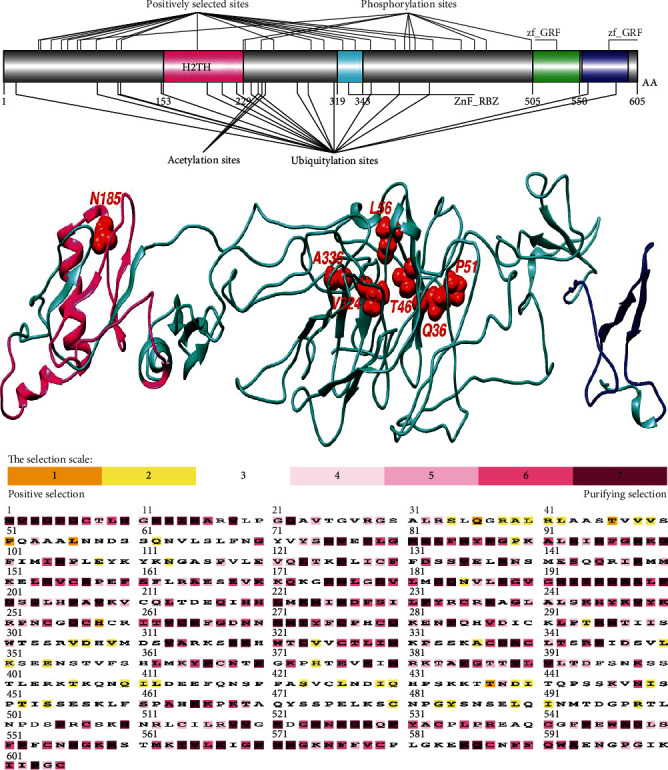
Illustration of protein domain structures using DOG 1.0 illustrator. The phosphorylation sites of the NEIL3 protein are shown in the molecular structure and conserved domain analyses. Positively selected amino acid sites were found in conserved domains, especially the H2TH domain. Positively identified locations were drawn into the 3D structure using human NEIL3 as a reference. Selecton analyses of human NEIL3 are color-coded and compared to sequences from aligned nucleotide coding sequences. Yellow and brown highlights represent positive selection, the neutral selection is represented by grey and white highlights, and purple highlights on codons represent purifying selection.

Positive selection sites were found in the H2TH domain (helix-2turn-helix domain), a DNA-binding domain of the NEIL studied. Positive selection was found in the Fapy-DNA-glyco and H2TH domains of human NEIL1 and the H2TH domains of NEIL2 and NEIL3 (Table 1 and Figures 1–3). The H2TH domain (helix-2turn-helix domain) is a DNA-binding domain found in DNA glycosylase/AP lyase enzymes involved in base excision repair of DNA damaged by oxidation or mutagenesis chemicals. It was shown that sequence differences in the H2TH domain of human NEIL genes, particularly around conserved areas or motifs, are predominantly linked with DNA repair during transcription and operate preferentially on cytosine-derived lesions, notably 5-hydroxyuracil and 5-hydroxycytocil. We examined the residues under selective constraints in three genes to evaluate the impact of positive selection on the H2TH domain (Figures 1–3). We found five positively chosen sites (S50, Q67, Q69, R90, and G107) in the H2TH domain of NEIL1, six sites (V190, V251, A254, R256, T269, and P280) in the H2TH domain of NEIL2, and just one site (N187) in the H2TH domain of NEIL3 protein. Positively chosen locations within or near the conserved motifs necessary for lyase activity induce nicks in the DNA strand, cleaving the DNA backbone to form a single-strand break at the site of the deleted base containing both 3′ and 5′-phosphates. Motifs involved in H2TH activities are conserved among NEIL proteins, as evidenced by numerous sequence alignments of human sequences (Figures 1–3).

We identified genes that are under positive selection across vertebrate species using a variety of site models. We tested alternative models for the dataset using the phylogenetic tree as input data. We used probability analysis to compare several ratio-based models to find codons subjected to positive selection in linked genes. The parameters linked with gene selection in 51 species were calculated using the codeml program, and the two sets of models (M1a vs. M2a and M7 vs. M8) were used to probe positive selection. The likelihood ratio test (LRT) value of 0 (*p* > 0.05) indicated that the NEIL1 gene test was not significant in M1a-M2a. However, the likelihood ratio test (LRT) value of 6.54 indicated that the NEIL1 gene test was significant in M7-M8. According to the test findings for positive selection model M8, the NEIL1 gene showed signs of positive selection, which means that M8 was accepted, whereas M7 was rejected (*p* > 0.05). The NEB and BEB analysis revealed that the codon positions in the NEIL2 gene showed positive selection at 95% and 99% probabilities, respectively. However, models M1a vs. M2a and M7 vs. M8 were very significant for the NEIL2 gene, with LRT values of 1.48 and 9.16, respectively (Table 1).

**Figure 4:**
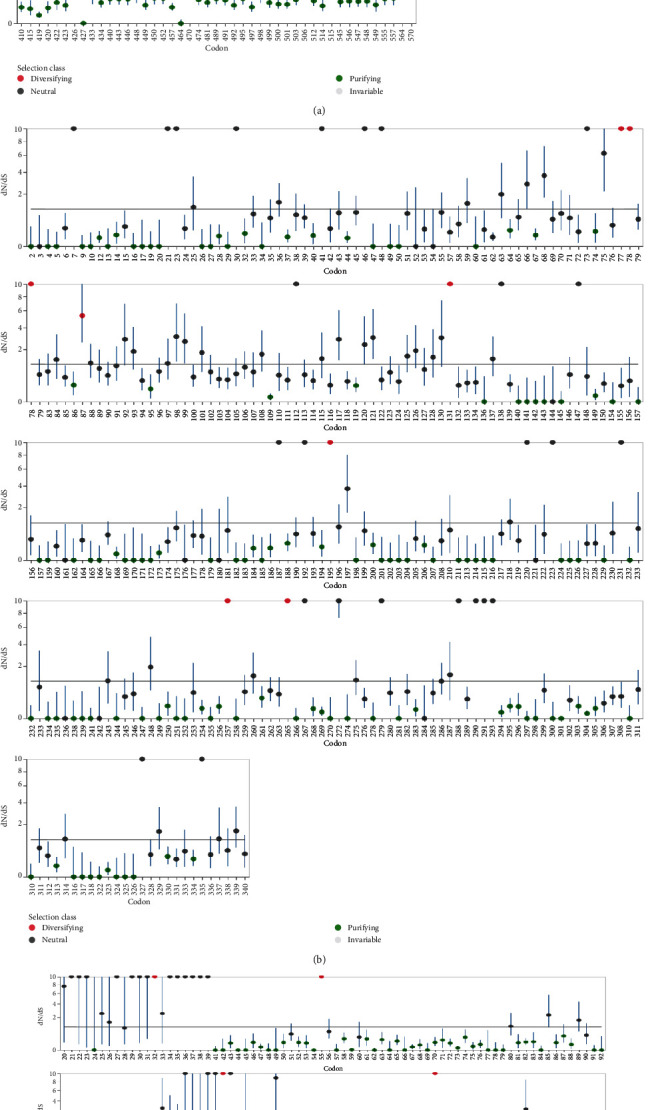
(a) Maximum likelihood estimations of dN/dS at each site of NEIL1, together with estimated profile confidence intervals. The dN/dS = 1 (neutrality) is depicted as a horizontal gray line. Boundaries between partitions (if present) are shown as vertical dashed lines. Statistical significance is evaluated based on the asymptotic *χ*2 distribution. This analysis includes site to site synonymous rate variation. Profile approximate confidence intervals for site-level dN/dS ratios have been computed. (b) Maximum likelihood estimations of dN/dS at each site of NEIL2, together with estimated profile confidence intervals. The dN/dS = 1 (neutrality) is depicted as a horizontal gray line. Boundaries between partitions (if present) are shown as vertical dashed lines. Statistical significance is evaluated based on the asymptotic *χ*2 distribution. This analysis includes site to site synonymous rate variation. Profile approximate confidence intervals for site-level dN/dS ratios have been computed. (c) Maximum likelihood estimations of dN/dS at each site of NEIL3, together with estimated profile confidence intervals. The dN/dS = 1 (neutrality) is depicted as a horizontal gray line. Boundaries between partitions (if present) are shown as vertical dashed lines. Statistical significance is evaluated based on the asymptotic *χ*2 distribution. This analysis includes site to site synonymous rate variation. Profile approximate confidence intervals for site-level dN/dS ratios have been computed.

Using SLAC, MEME, and FEL analyses, we analyzed the global values to identify the signals of positive selection during the evolutionary process (Figures 4(a)–4(c)). Our findings showed positive evolutionary selection in vertebrates' NEIL1, NEIL2, and NEIL3 genes. We used the Bayesian technique to identify the locations under selective pressure by computing the posterior probability for each codon. Diversifying selection with higher nonsynonymous/synonymous substitution rates is more likely at sites with higher probability than at locations with lower probabilities (Table 2). Using BEB analysis, we discovered multiple locations in the NEIL1 protein under positive selection with a high posterior probability of 95%. We confirmed positive selection by analyzing the results of PAML using the dataset in the Selecton server, which identifies adaptive selection at specific spots in the protein. The MEC model was used to detect the substitution rates. The results revealed that adaptive selection occurred at several amino acid positions in NEIL1, NEIL2, and NEIL3, among other proteins (Figures 1–3).

### 3.2. Pathway and Process Enrichment Analysis

**Figure 5:**
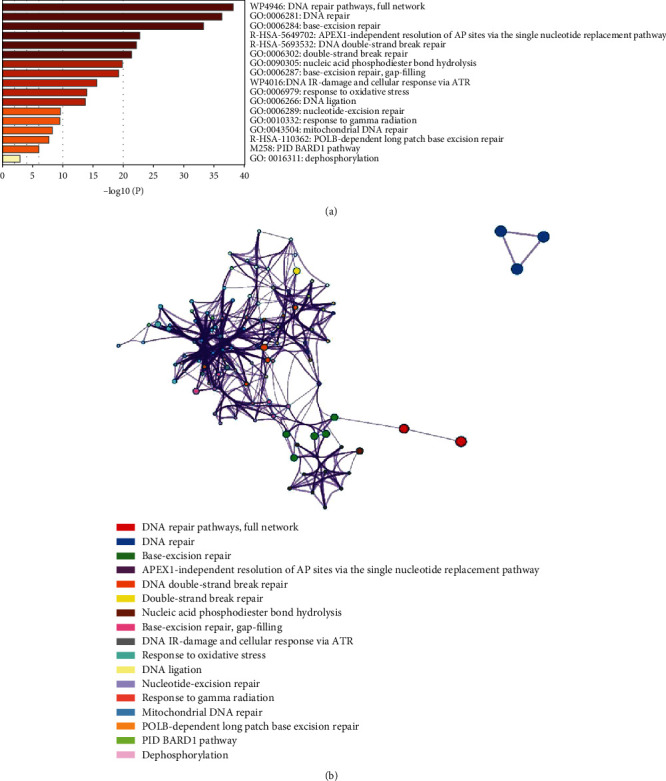
The common target genes of DNA repair pathways. GO annotation and KEGG were used to analyze these target genes.

Pathway and process enrichment analyses have been performed for each gene list using the following ontology sources: KEGG pathway, GO biological processes, reactome gene sets, PANTHER pathway, and WikiPathways. The enrichment background has been constructed from all of the genes in the genome. GO and KEGG pathway enrichment analyses were carried out on the data to get insight into the functions of the differentially expressed genes. At various periods, GO annotation indicated that biological processes and molecular activities linked to DNA repair pathways and base excision repair functions were enriched among the genes, indicating that the genes were involved in these processes and functions (Figures 5(a) and 5(b) and Table 3). Genes associated with oxidative stress responses and DNA repair were only significantly enriched in biological processes than their normal expression (Figures 5(a) and 5(b)). The cluster is represented by the statistically most significant keyword inside the cluster. To further visualize the links between the terms, a subclass of terms was selected and displayed as a linkage plot, in which the terms with a resemblance greater than 0.3 are connected by edges. We choose the GO terms with the best *p* values from each of the twenty clusters, with the constraint that there be no more than 15 terms per cluster and a total of 250 terms. The network is shown using Cytoscape, with each node representing an enhanced phrase and colored according to its cluster (Figure 5(b)). Eleven GO keywords were found to be enriched in both genotypes: DNA repair process, DNA repair pathways, full network, DNA repair, base-excision repair, replacement pathway, DNA double-strand break repair, double-strand break repair, hydrolysis, base-excision repair, response via ATR, response to oxidative stress, DNA ligation, and nucleotide-excision repair were detected and enriched (GO:0006281, GO:0006284, GO:0006302, GO:0090305, GO:0006287, GO:0006979, GO:0006266, GO:0006289, GO:0010332, and GO:0043504). DEGs in each of the three reaction stages were also characterized using GO term enrichment. DEGs in biological processes, molecular function, and cellular components were found in response to cold stress during the early reaction phase (Table 3).

### 3.3. Conservation and Coevolution Analysis

**Figure 6:**
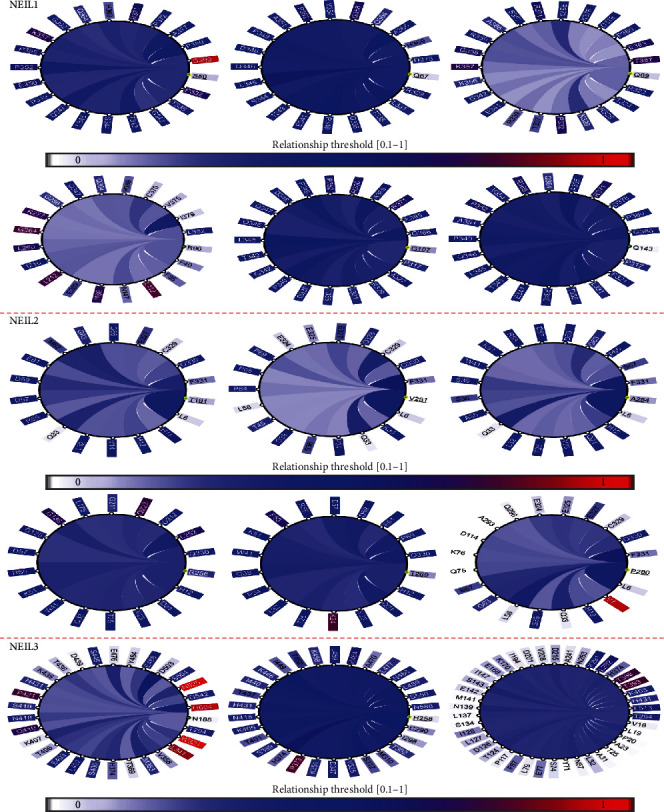
Coevolution of conserved domain residues. The circular connection diagram focuses on the residues with cutoffs of the NEIL1, NEIL2, and NEIL3 genes. The graphic shows the positions of amino acids in the protein. The colors of the curves denote covariance scores among spots.

**Figure 7:**
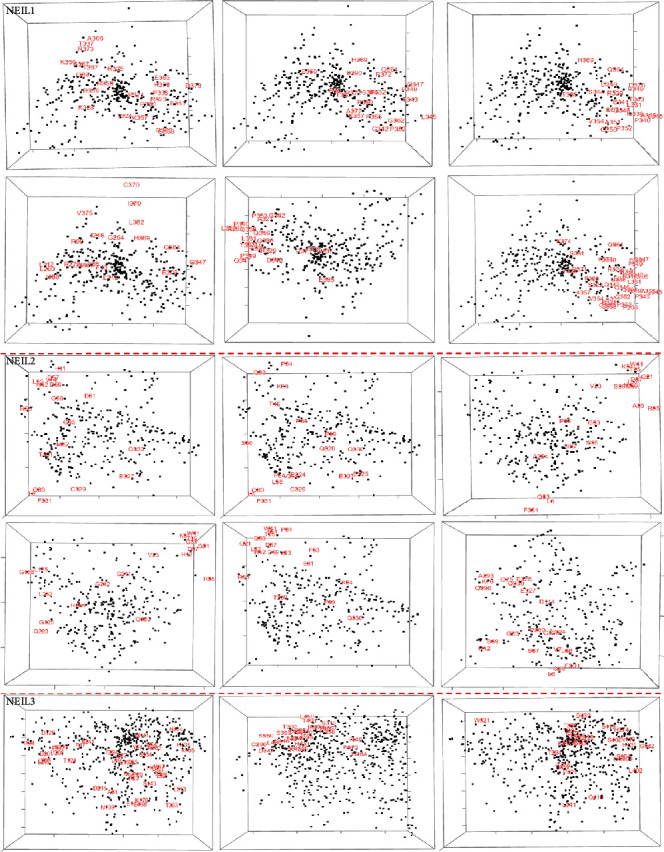
3D multidimensional scaling (MDS) scatterplots of covarying sites in NEIL1, NEIL2, and NEIL3 genes. (A) Highlighted red are the residues corresponding to the positively selected residue. Both black and red spots labeled showing the residues.

The functional and structural topographies of positively selected sites were assessed using coevolution analysis, which detects coordinated interactions between residues. In proteins, the coevolution of amino acid sites may be due to structural or functional links. We employed homologs of NEIL1, NEIL2, and NEIL3 as inputs in a coevolution analysis to identify different coevolving pairs formerly recognized under positive selection. A network diagram displaying the residues' connections was drawn to find a relationship between them (Figure 6). There is a correlation between amino acid residues and the extent of coevolutionary linkages between them. Nodes in a subnetwork containing only amino acids found in the protein's Fapy-DNA-glyco, H2TH, and zf-GRF domains had positively selected residues. NEIL1, NEIL2, and NEIL3 genes were shown to have coevolving probabilities according to Pearson correlation (*r*). This covariance study uncovered the spans and interactions between positively selected protein domain residues, as well as groups of functionally connected sites (Figure 7). The dynamic 3D panel allows you to zoom and rotate while labelling chosen residues. These findings support the idea that the NEIL protein sections that adhere to these conserved domains are structurally and functionally distinct.

## 4. Discussion

Animal genomes undergo significant evolutionary changes due to divergence, the adaptation of genetic material from gene duplication, divergent lineages, and epigenesist [48]. We use the findings of this phylogenetic study to infer the evolutionary history of repair pathways and the proteins that make them up and make predictions about the repair phenotypes of species with sequenced genomes [17]. Aside from that, we look at how evolutionary analysis can be used to study full genome sequences, how complete genome sequences may be used in evolutionary research, and the benefits of employing both the phylogenetic technique and evolutionary analysis [13]. Positive selection was discovered in the vertebrate NEIL genes. Positively chosen residues came in a variety of quantities and distributions. In the functional domains of the human NEIL1, NEIL2, and NEIL3 proteins, we discovered two positively chosen codons (Figures 1–3). The Nei-like glycosylases are crucial molecules involved in base excision repair in vertebrate genomes. It activates the range activity of substrates involved in DNA repair and maintenance. These proteins are involved in DNA replication coordination, detecting damage sites for repairing, and modifications in transcriptional regulation for expression of activated genes and recruitment of multifunctional protein factors for specific functions required for coordination in these activities [49]. The H2TH domain is required for downstream signaling in NEIL proteins since it is involved in DNA damage repair for accurate genome replication and transcript interpretation. Due to functional restrictions, residual with crucial functions may be under modest evolutionary pressure [50]. Core residues that make up the polar surface and hydrophobic core of the Fpg/Nei family of DNA glycosylases are conserved [51]. The Fapy domain of NEIL1 contained a smaller percentage of positively chosen codons and displayed more selection limitations than other NEIL proteins, as predicted [16].

**Figure 8:**
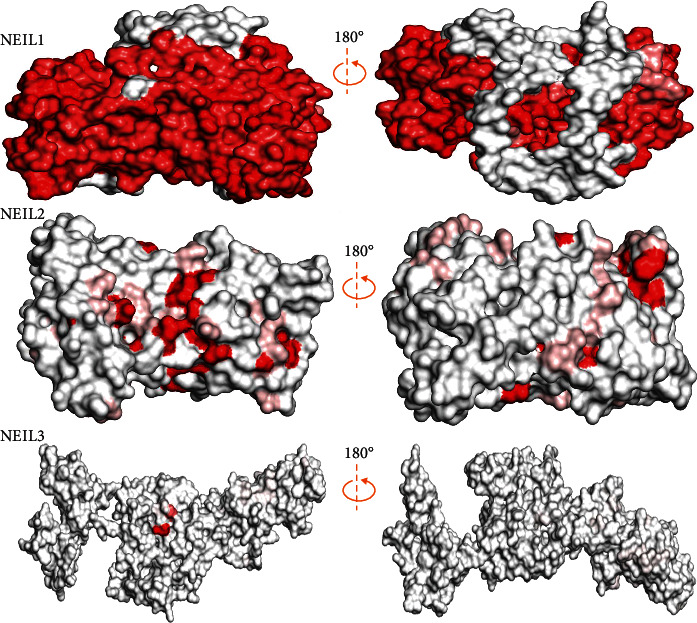
Surface representation of the human NEIL1, NEIL2, and NEIL3 proteins colored conferring to sequence conservation. The color ramping from white (low score) to red (high score) allows to quickly identify areas of weak and robust structural conservation of the proteins.

The study of genetics and evolutionary processes is required to understand the regulatory mechanisms of several physiologically relevant genes, such as the NEIL gene family in mammals. It was discovered that positive selection occurred at many locations in three genes across mammals, reptiles, and birds (Table 1). A gene's overall characteristic expression in these lineages was identical to that of genes that were not under selection or only selected in mammals [52]. According to our data, infections appear to represent a constant selection pressure throughout vertebrate clades. As we discovered, NEIL proteins had their amino acids buried and exposed, which did not affect H2TH's electrostatic distribution. Table 1 shows that residues (S50, Q67, R90, G107) of the NEIL2 H2TH domain that interacts with the NEIL3-H2TH domain are under positive selection. These residues are conserved across avians and humans (Figure 8), consistent with its crucial biological functions. In mammals, reptiles, and birds, three genes were identified under positive selection (Table 1). Because of this, we established standards similar to those of lineages where the selection was weak or merely positive in the vertebrates. Based on our findings, infections may exert a selective impact on several vertebrate taxa [53]. The first vertebrate phylogeny shifts in IRF, i.e., the discovery of an adaptive immune system, has been questioned by previous phylogenetic studies [54]. According to some theories, the IRF gene family originally consisted of two branches, which have been identified in all bilaterians, and cnidarians [55].

Analysis of protein alignments by MAFFT has shown an H2TH domain conserved in all three sequences of the NEIL1, NEIL2, and NEIL3 genes (Figures 2(b), 1(b), and 3(b)). The LRT values for NEIL1, NEIL2, and NEIL3 genes were 0, 1.48, and 0 for the M2 and M1 evolutionary models. Protein alterations in purifying selection zones suffer nonidentical shifts that are harmful to health and so have a low possibility of being fixed throughout evolution [56, 57]. This led to identifying amino acid residues having *ω* > 1 value in the next phase. Using the evolutionary model, several sites in NEIL1, NEIL2, and NEIL3 genes were identified under positive selection with LRT values 6.54, 9.16, and 0.48, respectively (Table 1). Our findings show that some locations

in other proteins subjected to strong positive selection have developed faster than the mature protein [22, 23]. As a result, dynamic selection induces a modification that aims to increase protein secretion effectiveness, as seen in the case of the NEIL proteins [58]. A branch site test was performed to determine which branches in vertebrate clades were being selectively selected for in the NEIL2 gene, and we found that just a few branches in the mammalian clade were being selectively selected. The NEIL3 gene exhibited signs of positive selection in both mammalian and avian clades. When we studied the NEIL genes, we identified positive selection in the majority of the vertebrate, which was surprising. We used the aBS-REL model to confirm our findings since branch site analysis can lead to ambiguous selection owing to multinucleotide mutations. The aBS-REL and site models both showed comparable patterns of selection. The findings show that the general selection trends we observed are correct [21, 23].

In general, these proteins in vertebrate lineages demonstrated little indication of positive selection, suggesting that genetic diversity is hampered by persistent selection pressure, especially in the avian clade. This might be linked to the idea that the gene's evolutionary history has been free of gene duplication occurrences [59]. Gene duplication is an evolutionary strategy that allows genomes to evolve in various ways. Positive selection occurs following a duplication event for other proteins, implying the selective pressure that supports genetic variety [60]. This relaxation was absent during the development of NEIL genes in avian and different vertebrate lineages, supporting Bayesian phylogenetic techniques. Our data show that purifying selection has traditionally determined the molecular evolution of NEIL genes in vertebrates. Proteins depend on coordinated interactions among their amino acid residues for structure and function [61]. As a result, identifying structural properties of positively chosen amino acid residues might be similar to detecting residues that covary throughout evolution. The structural or functional connections between amino acid residues inside proteins may have resulted in this coevolutionary relationship. Protein coevolution has previously been linked to protein constancy and intermolecular interactions [62, 63]. By measuring the dN/dS of mammalian Neil's sequences, we discovered that S50, Q67, Q69, R90, and G107 in the H2TH domain of NEIL1, six sites (V190, V251, A254, R256, T269, and P280) in the H2TH domain of NEIL2, and just one site (N187) in the H2TH domain of NEIL3 protein may play essential roles in several of the previously reported activities (Figures 1–3). The domains of NEIL1, NEIL2, and NEIL3 are largely conserved among vertebrates, suggesting that their functions are not duplicated and that this selection pressure stems from NEIL specialization for base excision repair pathways [49]. Proteins that interact directly with other molecules, on the other hand, have a higher probability of adapting to each other's evolutionary changes [64]. In the context of their initial function, NEIL genes may have experienced such coevolutionary features [65].

As a result, we used several sequence alignments obtained for NEIL1, NEIL2, and NEIL3 homologs to undertake a coevolution study, and we discovered positively chosen coevolving residues with significant variability (Figure 6). These findings show that the areas matching to domains in NEIL proteins are structure-function modules. Within H2Th and ZF-GRF domains, there were only a few adaptive substitutions, indicating a stable selection pattern throughout evolution. There has recently been an increase in the number of species with whole-genome sequences, allowing researchers to compare and contrast their DNA repair systems and discover how different repair genes and functional pathways have evolved [13]. It appears that the repair process is very variable among species, as evidenced by a worldwide comparison of DNA repair proteins based on all known genome sequences from microbes, archaea, and eukaryotes [66]. The unique gene insertions are commonly polymorphic (present or absent) at orthologous loci, making them highly useful genetic markers that may be employed in clustering studies, animal evolutionary history, population structure, and demography [67]. As a whole, these elements are known to affect the genome in a wide range of ways, including increasing the size of genome and instability, epigenetic regulation, and RNA editing [68]. Previously, [69] demonstrated significant structural conservation in the two-domain architecture, despite the considerable sequence divergence in the primarily b-strand-rich N-terminal domain. More study is needed to uniformly apply these potential indicators to different species because of genetic variation and locus distribution [70]. Species can develop their repair system through gene duplication and gene loss that often occurs in their populations [17]. Copy number variations of DNA repair genes will result from the species-specific history of gene duplication and loss, which will have a profound effect on organismal phenotypes such as mutation rates [16], lifespan [71], and adaptation to extreme environments [72, 73].

Furthermore, we reveal interesting data relating genetic changes in transcription activators and repressor elements from an evolutionary approach.

## 5. Conclusions

We think that the findings presented here may be used to begin experimental research of DNA repair in animals with entire genome sequences and better understand the evolution of proteins involved in DNA repair in the vertebrate genome. In conclusion, these comprehensive comparative assessments of DNA repair genes, particularly NEIL1, NEIL2, and NEIL3 in several species, revealed that these are the strong candidate genes related to tree lifetime; endogenous and external stressors can cause single-strand breaks bulky lesions, which play a critical role in DNA damage repair. As a result, our research may provide a platform on which to build future studies examining the connections between DNA repair and vertebrate species lifespan. Systematic comparative genome investigations will give vital insights to elucidate the links between DNA repair and lifespan development in many creatures when genome sequences of increasingly varied animal species become accessible. The present findings revealed that the conservation of dynamics as a component of a protein fold might have implications that go beyond enzyme catalysis in the future. The limitation of this study is that we used a limited number of species for our analyses may not be sufficient. Further analysis can be performed using genome sequencing technology from a large number of species available in future. Applying our analyses to the larger set of data will uncover the adaptive evolution of gene families involved in cellular longevity.

## Figures and Tables

**Table 1:** Results of positive selection tests for NEIL genes.

Gene	Models	lnL	LRT	PAML	SLAC	FUBAR	MEME
NEIL1	M1a	-11259.31	0	89,90,107,117,143,367,405,471	67, 107, 143, 405, 408, 444	67, 117, 143, 251, 367, 471	90, 117, 143, 405, 471
	M2a	-11259.31					
	M7	-11180.39	6.54				
	M8	-11177.12					
NEIL2	M1a	-7282.58	1.48	65, 67, 84, 94, 103, 105, 116, 191, 256, 301, 329	103, 191, 236, 329	65, 103, 116, 154, 191, 301, 329	103, 105, 191, 256, 329
	M2a	-7281.84					
	M7	-7273.27	9.16				
	M8	-7268.69					
NEIL3	M1a	-17093.12	0	36, 46, 51, 56, 62, 89, 108, 113, 185, 258, 309, 336, 437	46, 62, 108, 113, 185, 309	36, 51, 62, 89, 113, 185, 309, 437	51, 62, 113, 185, 309, 437
	M2a	-17093.12					
	M7	-16962.42	0.48				
	M8	-16962.18					

**Table 2:** Detailed site-by-site results from the FEL analysis. Statistical significance is evaluated based on the asymptotic *χ*2 distribution. This analysis includes site to site synonymous rate variation. Profile approximate confidence intervals for site-level dN/dS ratios have been computed.

Gene	Codons	*α*	*β*	*α* = *β*	LRT	*P* value	Total branch length	dN/dS LB	dN/dS MLE	dN/dS UB	Selection type
NEIL1	114	0.00	1.519	34.71	2.169	0.1408	296.96	3,827.26	10,00	10,000	Diversifying
	143	0.00	0.538	0.405	3.391	0.0655	3.464	7,865.16	10,00	10,000	Diversifying
	268	0.00	1.002	0.612	1.897	0.1684	5.234	3,827.16	10,00	10,000	Diversifying
	408	0.00	1.123	0.713	4.603	0.0319	6.100	8,626.24	10,00	10,000	Diversifying
	419	0.00	0.495	0.321	1.662	0.1973	2.746	3,826.84	10,00	10,000	Diversifying
	448	0.22	0.885	0.551	1.873	0.1711	4.711	1.347	4.058	13.044	Diversifying
	455	0.24	1.397	0.855	2.04	0.1533	7.314	2.417	5.894	15.896	Diversifying
NEIL2	77	0.00	0.682	0.404	4.036	0.0445	1.437	6,186.34	10,00	10,000	Diversifying
	78	0.00	2.266	1.678	4.706	0.0301	5.968	8,078.11	10,00	10,000	Diversifying
	87	0.70	3.768	2.879	3.02	0.0822	10.24	2.525	5.348	11.869	Diversifying
	131	0.00	1.33	0.851	5.615	0.0178	3.027	7,599.97	10,00	10,000	Diversifying
	195	0.00	0.871	0.605	3.42	0.0644	2.153	6,810.13	10,00	10,000	Diversifying
	257	0.00	0.857	0.558	4.19	0.0407	1.984	6,810.14	10,00	10,000	Diversifying
	265	0.00	0.648	0.415	3.472	0.0624	1.477	6,186.32	10,00	10,000	Diversifying
NEIL3	32	0.00	1.281	10000	1.093	0.2957	85553.36	3,827.32	10,00	10,000	Diversifying
	55	0.00	0.519	0.406	1.487	0.2227	3.469	7,599.63	10,00	10,000	Diversifying
	114	0.00	1.519	55.08	2.169	0.1408	471.262	3,827.26	10,00	10,000	Diversifying
	143	0.00	0.538	0.405	3.391	0.0655	3.464	7,865.16	10,00	10,000	Diversifying
	268	0.00	1.002	0.612	1.897	0.1684	5.234	3,827.16	10,00	10,000	Diversifying
	271	0.00	0.694	0.281	1.615	0.2038	2.407	1,464.80	10,00	10,000	Diversifying
	408	0.00	1.123	0.713	4.603	0.0319	6.100	8,626.24	10,00	10,000	Diversifying
	419	0.00	0.495	0.321	1.662	0.1973	2.746	3,826.84	10,00	10,000	Diversifying
	444	0.74	4.696	3.046	1.594	0.2067	26.059	2.105	6.370	47.341	Diversifying
	448	0.22	0.885	0.551	1.873	0.1711	4.711	1.347	4.058	13.044	Diversifying
	455	0.24	1.397	0.855	2.04	0.1533	7.314	2.417	5.894	15.896	Diversifying
	472	0.57	2.486	1.641	1.205	0.2724	14.039	1.59	4.361	13.636	Diversifying

*α*: synonymous substitution rate at a site; *β*: nonsynonymous substitution rate at a site; *α* = *β*: the rate estimate under the neutral model; LRT: likelihood ratio test statistic for beta = alpha, versus beta &neq; alpha; *P* value: asymptotic *P* value for evidence of selection, i.e., beta &neq; alpha; total branch length: the total length of branches contributing to inference at site used to scale dN-dS; dN/dS LB: 95% profile likelihood CI lower bound for dN/dS; dN/dS MLE: point estimate for site dN/dS; dN/dS UB: 95% profile likelihood CI upper bound for dN/dS.

**Table 3:** Go terms and clusters with enriched keywords (one per cluster). This is the number of genes in the user-supplied lists that belong to the ontology term.

GO	Category	Description	Count	%	*Log*10(*P*)	*Log*10(*q*)
WP4946	WikiPathways	DNA repair pathways, full network	17	85	-38.21	-33.87
GO:0006281	GO biological processes	DNA repair	20	100	-36.36	-32.31
GO:0006284	GO biological processes	Base-excision repair	13	65	-33.31	-29.57
R-HSA-5649702	Reactome gene sets	Replacement pathway	7	35	-22.77	-19.46
R-HSA-5693532	Reactome gene sets	DNA double-strand break repair	12	60	-22.18	-18.91
GO:0006302	GO biological processes	Double-strand break repair	12	60	-21.4	-18.17
GO:0090305	GO biological processes	Hydrolysis	12	60	-19.86	-16.66
GO:0006287	GO biological processes	Base-excision repair	7	35	-19.23	-16.06
WP4016	WikiPathways	Response via ATR	8	40	-15.64	-12.52
GO:0006979	GO biological processes	Response to oxidative stress	10	50	-14.01	-10.94
GO:0006266	GO biological processes	DNA ligation	5	25	-13.73	-10.68
GO:0006289	GO biological processes	Nucleotide-excision repair	5	25	-9.6	-6.86
GO:0010332	GO biological processes	Response to gamma radiation	5	25	-9.56	-6.83
GO:0043504	GO biological processes	Mitochondrial DNA repair	3	15	-8.3	-5.8
R-HSA-110362	Reactome gene sets	Excision repair	3	15	-7.68	-5.24
M258	Canonical pathways	PID BARD1 PATHWAY	3	15	-6.05	-3.78
GO:0016311	GO biological processes	Dephosphorylation	3	15	-2.97	-0.87

## Data Availability

Data will be available openly to the readers.

## Supplementary Materials

Supplementary Materials Supplementary material showing the list of vertebrate species and NCBI Genbank accession numbers for sequences used to build datasets for hypothesis testing of the NEIL genes. Table S1: List of vertebrate species and NCBI Genbank accession numbers for sequences used to build datasets for hypothesis testing of the NEIL1 gene. Table S2: List of vertebrate species and NCBI Genbank accession numbers for sequences used to build datasets for hypothesis testing of the NEIL2 gene. Table S3: List of vertebrate species and NCBI Genbank accession numbers for sequences used to build datasets for hypothesis testing of the NEIL3 gene.
