# Supplementary material for: Structural and Evolutionary Adaptations of Nei-Like DNA Glycosylases Proteins Involved in Base Excision Repair of Oxidative DNA Damage in Vertebrates
Source: Oxid Med Cell Longev. 2022 Apr 4;2022:1144387. doi: 10.1155/2022/1144387 (PMC9001079; doi:10.1155/2022/1144387)
Supplement: Supplementary Materials — Supplementary material showing the list of vertebrate species and NCBI Genbank accession numbers for sequences used to build datasets for hypothesis testing of the NEIL genes. Table S1: List of vertebrate species and NCBI Genbank accession numbers for sequences used to build datasets for hypothesis testing of the NEIL1 gene. Table S2: List of vertebrate species and NCBI Genbank accession numbers for sequences used to build datasets for hypothesis testing of the NEIL2 gene. Table S3: List of vertebrate species and NCBI Genbank accession numbers for sequences used to build datasets for hypothesis testing of the NEIL3 gene. [file 1144387.f1.docx]

**Supplementary material showing the list of vertebrate species and NCBI Genbank accession numbers for sequences used to build datasets for hypothesis testing of the NEIL genes.**

**Table S1.** List of vertebrate species and NCBI Genbank accession numbers for sequences used to build datasets for hypothesis testing of the NEIL1 gene.

| **Scientific name** | **Common name** | **Accession number** |
| --- | --- | --- |
| *Homo sapiens* | Human | ENSG00000140398 |
| *Rattus norvegicus* | Norway rat | NM_001025754.1 |
| *Balaena mysticetus* | Bowhead whale | MZ055370.1 |
| *Gambusia affinis* | Western mosquitofish | XM_044123752.1 |
| *Danio rerio* | Zebrafish | NM_200283.2 |
| *Solea senegalensis* | Senegalese sole | XM_044035839.1 |
| *Gallus gallus* | Chicken | NM_001080876.3 |
| *Puntigrus tetrazona* | Sumatra barb | XM_043228357.1 |
| *Chrysemys picta bellii* | Western painted turtle | XM_042862046.1 |
| *Centrocercus urophasianus* | Greater sage-grouse | XM_042821208.1 |
| *Orycteropus afer afer* | Aardvark | XM_007948944.1 |
| *Oncorhynchus tshawytscha* | Chinook salmon | XM_042327171.1 |
| *Alosa sapidissima* | American shad | XM_042110289.1 |
| *Melanotaenia boesemani* | Boeseman's rainbowfish | XM_041996418.1 |
| *Chelmon rostratus* | Copperband butterflyfish | XM_041938629.1 |
| *Coregonus clupeaformis* | Lake whitefish | XM_041849686.1 |
| *Taeniopygia guttata* | Zebra finch | XM_030281287.3 |
| *Aquila chrysaetos chrysaetos* | Golden Eagle | XM_030015030.2 |
| *Toxotes jaculatrix* | Banded archerfish | XM_041037520.1 |
| *Amblyraja radiata* | Thorny skate | XM_033014810.1 |
| *Ornithorhynchus anatinus* | Platypus | XM_029066048.1 |
| *Pteropus giganteus* | Indian flying fox | XM_039856699.1 |
| *Pimephales promelas* | Fathead minnow | XM_039647936.1 |
| *Corvus cornix cornix* | Hooded crow | XM_019293540.2 |
| *Saimiri boliviensis boliviensis* | Bolivian squirrel monkey | XM_003942997.3 |
| *Pipra filicauda* | Wire-tailed manakin | XM_027712143.2 |
| *Motacilla alba alba* | White wagtail | XM_038146961.1 |
| *Sebastes umbrosus* | Honeycomb rockfish | XM_037767515.1 |
| *Pygocentrus nattereri* | Red-bellied piranha | XM_017708367.2 |
| *Acanthopagrus latus* | Yellowfin seabream | XM_037106551.1 |
| *Colossoma macropomum* | Tambaqui | XM_036576042.1 |
| *Ochotona princeps* | American pika | XM_004594693.1 |
| *Molothrus ater* | Brown-headed Cowbird | XM_036389995.1 |
| *Fundulus heteroclitus* | Mummichog | XM_012857044.3 |
| *Egretta garzetta* | Little egret | XM_035899513.1 |
| *Zalophus californianus* | California sea lion | XM_027571244.2 |
| *Cygnus atratus* | Black swan | XM_035553821.1 |
| *Thalassophryne amazonica* | Venomous toadfishes | XM_034176444.1 |
| *Epinephelus lanceolatus* | Giant grouper | XM_033635163.1 |
| *Mastacembelus armatus* | Zig-zag eel | XM_026311813.1 |
| *Trachypithecus francoisi* | Francois's langur | XM_033190973.1 |
| *Lacerta agilis* | Sand lizard | XM_033160713.1 |
| *Chiroxiphia lanceolata* | Lance-tailed manakin | XM_032700660.1 |
| *Xiphophorus hellerii* | Green swordtail | XM_032583121.1 |
| *Coturnix japonica* | Japanese quail | XM_015872609.2 |
| *Phoca vitulina* | Harbor seal | XM_032417696.1 |
| *Aythya fuligula* | Tufted duck | XM_032195068.1 |
| *Sarcophilus harrisii* | Tasmanian devil | XM_031956584.1 |
| *Piliocolobus tephrosceles* | Ugandan red Colobus | XM_023196652.3 |
| *Xenopus tropicalis* | Tropical clawed frog | NM_001122798.1 |

**Table S2.** List of vertebrate species and NCBI Genbank accession numbers for sequences used to build datasets for hypothesis testing of the NEIL2 gene.

| **Scientific name** | **Common name** | **Accession number** |
| --- | --- | --- |
| *Homo sapiens* | Human | ENSG00000154328 |
| *Mus musculus* | House mouse | NM_201610.2 |
| *Bos taurus* | Cattle | NM_001013003.1 |
| *Pongo abelii* | Sumatran orangutan | NM_001132242.1 |
| *Gracilinanus agilis* | Agile Gracile Mouse Opossum | XM_044660912.1 |
| *Protopterus annectens* | West African lungfish | XM_044062380.1 |
| *Rattus norvegicus* | Norway rat | NM_001107270.1 |
| *Varanus komodoensis* | Komodo dragon | XM_044450323.1 |
| *Dromiciops gliroides* | Monito del monte | XM_043988834.1 |
| *Dermochelys coriacea* | Leatherback sea turtle | XM_038397199.2 |
| *Tyto alba* | Barn owl | XM_009971062.3 |
| *Chrysemys picta bellii* | Western painted turtle | XM_005290680.3 |
| *Orycteropus afer afer* | Aardvark | XM_007938764.1 |
| *Dipodomys spectabilis* | Banner-tailed kangaroo rat | XM_042699862.1 |
| *Sceloporus undulatus* | Fence lizard | XM_042444976.1 |
| *Peromyscus maniculatus bairdii* | Prairie deer mouse | XM_006993002.3 |
| *Ovis aries* | Sheep | XM_004004449.4 |
| *Sturnira hondurensis* | Choco yellow-shouldered bat | XM_037042172.1 |
| *Corvus kubaryi* | Mariana crow | XM_042032799.1 |
| *Vulpes lagopus* | Arctic fox | XM_041767978.1 |
| *Taeniopygia guttata* | Zebra finch | XM_012572938.4 |
| *Microtus oregoni* | Creeping vole | XM_041674193.1 |
| *Pyrgilauda ruficollis* | Rufous-necked snowfinch | XM_041480978.1 |
| *Onychostruthus taczanowskii* | White-rumped snowfinch | XM_041426210.1 |
| *Aquila chrysaetos chrysaetos* | Golden Eagle | XM_030037037.2 |
| *Falco naumanni* | Lesser kestrel | XM_040598453.1 |
| *Amblyraja radiata* | Thorny skate | XM_033026361.1 |
| *Rana temporaria* | Common frog | XM_040348352.1 |
| *Ictidomys tridecemlineatus* | Thirteen-lined ground squirrel | XM_040292093.1 |
| *Corvus cornix cornix* | Hooded crow | XM_010411406.4 |
| *Saimiri boliviensis boliviensis* | Bolivian squirrel monkey | XM_039461125.1 |
| *Canis lupus familiaris* | Dog | XM_543204.7 |
| *Tachyglossus aculeatus* | Australian echidna | XM_038771533.1 |
| *Motacilla alba alba* | White Wagtail | XM_038133172.1 |
| *Choloepus didactylus* | Southern two-toed sloth | XM_037812889.1 |
| *Falco rusticolus* | Gyrfalcon | XM_037392079.1 |
| *Peromyscus leucopus* | White-footed mouse | XM_028884120.2 |
| *Artibeus jamaicensis* | Jamaican fruit-eating bat | XM_037145423.1 |
| *Balaenoptera musculus* | Blue whale | XM_036856652.1 |
| *Trichosurus vulpecula* | Common brushtail | XM_036752766.1 |
| *Ochotona princeps* | American pika | XM_004579299.1 |
| *Molothrus ater* | Brown-headed Cowbird | XM_036381155.1 |
| *Molossus molossus* | Pallas's mastiff bat | XM_036240414.1 |
| *Onychomys torridus* | Southern grasshopper mouse | XM_036199574.1 |
| *Phyllostomus discolor* | Pale spear-nosed bat | XM_036032015.1 |
| *Egretta garzetta* | Little egret | XM_009639929.2 |
| *Canis lupus dingo* | Dingo | XM_025462903.2 |
| *Zalophus californianus* | California sea lion | XM_027599001.2 |
| *Cygnus atratus* | Black swan | XM_035563706.1 |
| *Cricetulus griseus* | Chinese hamster | XM_035452257.1 |
| *Gallus gallus* | Chicken | NM_001277833.1 |

**Table S3.** List of vertebrate species and NCBI Genbank accession numbers for sequences used to build datasets for hypothesis testing of the NEIL3 gene.

| **Scientific name** | **Common name** | **Accession number** |
| --- | --- | --- |
| *Mus musculus* | House mouse | NM_146208.2 |
| *Homo sapiens* | Human | ENSG00000109674 |
| *Rattus norvegicus* | Norway rat | NM_001170346.1 |
| *Xenopus tropicalis* | Tropical clawed frog | NM_001017201.2 |
| *Bos taurus* | Cattle | NM_001034490.2 |
| *Danio rerio* | Zebrafish | NM_001007335.1 |
| *Gracilinanus agilis* | Agile Gracile Mouse Opossum | XM_044681663.1 |
| *Ursus arctos horribilis* | Grizzly bear | XM_044387583.1 |
| *Varanus komodoensis* | Komodo dragon | XM_044430849.1 |
| *Thunnus albacares* | Yellowfin tuna | XM_044364023.1 |
| *Gambusia affinis* | Western mosquitofish | XM_044127071.1 |
| *Solea senegalensis* | Senegalese sole | XM_044040137.1 |
| *Cervus elaphus* | Red deer | XM_043893855.1 |
| *Prionailurus bengalensis* | Leopard cat | XM_043557922.1 |
| *Puntigrus tetrazona* | Sumatra barb | XM_043256442.1 |
| *Centrocercus urophasianus* | Greater sage-grouse | XM_042812495.1 |
| *Nematolebias whitei* | Rio pearl fish | XM_037675469.1 |
| *Cyprinus carpio* | Common carp | XM_042738293.1 |
| *Clupea harengus* | Atlantic herring | XM_012834279.3 |
| *Dipodomys spectabilis* | Banner-tailed kangaroo rat | XM_042701161.1 |
| *Plectropomus leopardus* | Leopard coralgrouper | XM_042493599.1 |
| *Thunnus maccoyii* | Southern blue fin tuna | XM_042419050.1 |
| *Callorhinchus milii* | Elephant shark | XM_007895922.2 |
| *Oncorhynchus tshawytscha* | Chinook salmon | XM_024383116.2 |
| *Ovis aries* | Sheep | XM_015104551.3 |
| *Haplochromis burtoni* | Burton's mouth brooder | XM_005919410.3 |
| *Corvus kubaryi* | Mariana crow | XM_042014876.1 |
| *Melanotaenia boesemani* | Boeseman's rainbowfish | XM_041990662.1 |
| *Chelmon rostratus* | Copperband butterflyfish | XM_041944162.1 |
| *Coregonus clupeaformis* | Lake whitefish | XM_041850577.1 |
| *Cheilinus undulatus* | Humphead wrasse | XM_041797302.1 |
| *Microtus oregoni* | Creeping vole | XM_041673909.1 |
| *Pyrgilauda ruficollis* | Rufous-necked snow finch | XM_041480164.1 |
| *Betta splendens* | Siamese fighting fish | XM_029165345.2 |
| *Mesocricetus auratus* | Golden hamster | XM_005066686.4 |
| *Ursus maritimus* | Polar bear | XM_040643407.1 |
| *Falco naumanni* | Lesser kestrel | XM_040599575.1 |
| *Puma yagouaroundi* | Jaguarundi | XM_040489874.1 |
| *Oryx dammah* | Scimitar-horned oryx | XM_040243240.1 |
| *Simochromis diagramma* | Perch | XM_040010373.1 |
| *Perca fluviatilis* | European perch | XM_039813901.1 |
| *Oreochromis aureus* | Blue tilapia | XM_031758851.2 |
| *Scyliorhinus canicula* | Smaller spotted catshark | XM_038805753.1 |
| *Micropterus salmoides* | Largemouth bass | XM_038699653.1 |
| *Cyprinodon tularosa* | The White Sands pupfish | XM_038269100.1 |
| *Choloepus didactylus* | Southern two-toed sloth | XM_037829110.1 |
| *Cebus imitator* | Panamanian white-faced capuchin | XM_017542205.2 |
| *Pygocentrus nattereri* | Red-bellied piranha | XM_017693376.2 |
| *Talpa occidentalis* | Iberian mole | XM_037526471.1 |
| *Pungitius pungitius* | Ninespine stickleback | XM_037462030.1 |
